# Exploring the needs and possibilities of physicians’ continuing professional development - An explorative qualitative study in a Chinese primary care context

**DOI:** 10.1371/journal.pone.0202635

**Published:** 2018-08-16

**Authors:** Egui Zhu, Uno Fors, Åsa Smedberg

**Affiliations:** 1 Department of Learning, Informatics, Management and Ethics, Karolinska Institutet, Stockholm, Sweden; 2 Department of Computer and Systems Sciences, Stockholm University, Stockholm, Sweden; Public Library of Science, UNITED KINGDOM

## Abstract

**Background:**

One component of the 2009 Chinese health care reform plan is to train general practitioners to improve the delivery of primary care services. This continuing professional development is expected to further improve the physicians’ competencies to be general practitioners in primary care. Augmented reality–a combination of virtual information and the real environment–may enhance general practitioners’ continuing professional development by allowing their learning experiences to overlap with their workplace practice.

**Objective:**

To explore the needs, opportunities, and challenges involved in continuing professional development for Chinese physicians becoming competent general practitioners within primary care, with a special focus on the possibilities of applying augmented reality.

**Methods:**

This study used a qualitative approach with semi-structured face-to-face interviews. Two managers and thirteen physicians (from four community health centers and stations) participated. The data were analyzed using a thematic inductive analysis approach.

**Results:**

Based on our interviews, most of the physicians were not fully trained as general practitioners but still assumed the duties of that position; they were supposed to eventually become fully trained in line with the reforms of the Chinese primary care system. However, they reported a lack of in-service training opportunities to fulfill this goal. Even those who said that they had such opportunities perceived the efficacy of that training as being poor. The managers and most of the physicians reacted positively to the idea of using augmented reality in continuing professional development, and they suggested antibiotics treatment, surgery, and emergency care as learning areas in which augmented reality could be applied.

**Conclusions:**

Due to the Chinese reforms of the primary care system, both managers and the physicians themselves expect general practitioners to become qualified by engaging in continuing professional development. Both groups also regarded augmented reality as a potentially useful tool.

## Introduction

The 2009 Chinese health care reforms had the objective of building a system that would provide affordable and equitable basic health care for the whole population by 2020; these plans had several components. Yip et al (2012) summarized the plans and the recent progress toward the overall objective [1]. The core of the reform was to improve primary care by supporting community health centers or community health stations (CHCSs) in cities and township health centers in rural areas. In parallel, China initiated a program focused on essential medicines; one of its targets was to reduce the overprescription of antibiotics. China also put special emphasis on using training to improve the delivery of primary care services.

The Chinese government accompanied the implementation of the reform plan with increased funding for primary care providers to provide their service area with basic public health services. In addition, the government subsidized health insurance, especially for rural residents in the central and western China. In 2012, the government reported some positive developments related to these changes, stating that most of the goals related to the basic public health package had been reached.

In the Chinese health system, hospital dominance has been a significant challenge [2]. This is reflected in the government officials’ limited understanding of the role that competent primary care practitioners play in both managing health and disease and securing cost efficiency in the system [3]. Primary care also has somewhat of an image problem because of insufficiently trained physicians. In 2013, only 42.1% of licensed primary care physicians in urban areas had an undergraduate medical education; in rural areas, this figure was as low as 16.2% [4]. At the end of 2012, 1.02 million village doctors had received only vocational training [4]. The recent plan to strengthen primary care has been hampered by the population’s lack of trust in primary care practitioner and by the related tendency to turn directly to hospitals for elementary services.

In 2010, the Chinese Ministry of Health launched the National Health Protection Project to train 300,000 general practitioners (GPs) by 2020. That plan has been hampered by the lack of a primary care curriculum in most undergraduate medical programs [5]. Presently, only 13 out of 76 medical schools included in a recent survey train students in primary care and general practice; in addition, some of the schools that do offer such training have had problems recruiting students [6]. The Chinese government introduced in-service training in 2000 to qualify for hospital specialists as GPs; this program was reinforced in 2010 [7,8]. A GP license can be acquired by passing a national GP qualification exam or by successfully completing 500 hours of in-service GP training [9].

In reality, primary care physicians in China comprise a mixed group of medical practitioners: physicians participating in internships, residents in general practice, hospital specialists (with or without additional training in general practice), and specialists in both general practice and community medicine.

Globally, continuing professional development (CPD) throughout the career is essential to sustained professionalism, professional competence, and high-quality service. Given the challenges of Chinese primary care, organized CPD is necessary to meet primary care practitioners’ perceived needs; such training must be relevant enough to attract physicians to participate.

Scholars have suggested two initial steps for creating an effective CPD cycle: identifying why the CPD should be carried out and stating which learning methods should be used [10]. There is no standard CPD learning method, as it depends on the GPs’ individual needs [11]. Different countries have different CPD requirements [12]. The many ways to analyze GPs’ CPD needs include patient feedback, clinical experience, personal views, and previous CPD training [13]. The support of the managers and the organization as a whole is another important factor in the effectiveness of CPD [14]. Grant listed many learning methods for delivering CPD, including activities involving academic learning, meetings, and either practice- or technology-based learning [10].

The increased availability of various technologies could help GPs to control and enhance their learning and health care professional development [15,16]. Augmented reality (AR) is one such technology; AR has educational applications and could provide learning experiences that combine the real and virtual worlds [17]. AR thus gives GPs the opportunity to connect their in-practice learning experience with their CPD [18].

In AR, a physical real-world environment is augmented with virtual (computer-generated) information to illustrate various views to the user [19,20]. AR has the following characteristics: (1) It is used in conjunction with the surrounding real-world environment to provide an authentic and situated experience; (2) the physical environment surrounding the users is enhanced with virtual information that the users can interact with; and (3) users can indirectly experience their surroundings with senses that are enhanced through virtual information [21]. The first educational AR was developed for workplace learning [22], and it has been used in health care education since 2002 [23]. A more recent example is a mobile phone AR application that has been used to teach forensic medicine [24].

When integrating new technologies into the workplace, particularly for education, user acceptance is an important success factor [25]. Davis proposed a technology-acceptance model with user-motivation variables such as attitude toward use, perceived usefulness, and perceived ease of use [26]. Davis also suggested investigating technology acceptance in the early design stage so as to yield the greatest design flexibility within the limits of development costs. Doing so would help to reduce the number of user rejections. Although technology is expected to enhance learning, it can also change learners’ understanding of and expectations regarding the nature of learning [27]. However, rushing to use a technology without first properly addressing user acceptance can lead to problems such as low motivation to use the technology and a lack of significant effective uses [28].

To our knowledge, scholars have not explored the use of AR in CPD for GPs to a significant degree anywhere, and particularly not in China [29,30]. Consequently, our aim in this study was to explore the extent to which novel ways of organizing CPD can meet the needs of the various types of physicians who are presently employed as primary care practitioners in China, thus increasing their interest in joining CPD programs. More specifically, we set out to determine whether primary care physicians would accept AR as a learning method for CPD programs. We formulated two research questions:

RQ1: To what extent do managers and physicians perceive that current CPD programs meet their own expectations and fulfill the government’s requirements?

RQ2: Regarding in-service GP training, what are managers’ and physicians’ attitudes toward using AR in CPD?

## Methods

### Study setting

This study was conducted in Wuhan, China, which has been one of ten pilot cities in the national GP service program since 2012. At the time of the study, 466 of the 540 CHCSs in Wuhan were located within its seven main districts [31]. Only 200 GPs worked in Wuhan in 2012 [32]; this is far below the estimated need of more than 5,000 GPs (five per 10,000 people) according to the city’s population [33].

### Study design

To address the posed research questions, we chose a qualitative study design comprising interviews with two CHCSs managers (both women) and thirteen practicing physicians (ten women) from four CHCSs. To recruit participants, the first author (EZ) contacted the managers of seven CHCS in each of Wuhan’s seven main districts (as most CHCSs in that city are located in those districts). Two managers, who were directors and who were responsible for providing CPD to physicians there, agreed to participate, and they granted us access to recruit and interview the physicians at their workplaces. They suggested that physicians could be approached during night shifts, when the chance of finding available GPs was the highest and we recruited them using convenience sampling. Recruitment proved challenging, however, and the data collection phase was paused after only three physicians had participated. We decided to use snowball sampling as a complementary approach to more efficiently increase the number of participants and to create variety in their backgrounds and experiences, thus enriching the study’s insights. By maintaining contact with two of the physicians from the first phase, we managed to recruit additional participants for the main data collection, which took place in December 2014 and January 2015. At this time, recruitment was easier, as Wuhan’s pilot program for the national GP services had developed a bit more. In this period, we interviewed one of the participants who had acted as contact persons a second time; this enabled us to gain insight into recent developments. However, the other participant was not available during the second phase of data collection because she was on vacation. Saturation was reached after interviews with an additional ten physicians during their daytime shifts.

### Data collection

EZ carried out all the interviews in person, using a semi-structured interview guide to ensure that the research questions were covered (Table 1). Furthermore, to help the interviewees understand the nature of AR, the interviewer presented them with a free app, PlayAR Human Anatomy 4D for iPad (version 1.4.04, PlayAR Games, United States). The interviewer used interactive questioning and reflective commentary to enhance her credibility [34]. The interviews, which lasted 30 minutes on average, were audio recorded and transcribed verbatim in Mandarin.

**Table 1:** Interview questions for the managers and physicians.

Managers	Physicians
1) How many physicians are employed at your CHCS and what are their titles and degrees?	1) When did you graduate, and what subjects did you study?
2) What strategies does your CHCS use for the physicians’ CPD, and how are CPD performances perceived?	2) What is your role in the CHCS?
3) How many physicians join the GP in-service training each year?	3) How would you rate your CPD since graduation? And what activities do you consider effective?
4) How is the training structured of the GP in-service and how effective is it?	What challenges have you experienced in CPD?
4) What barriers have you experienced in the current CPD activities?	4) Do you plan to participate in future GP in-service trainings?
5) What are your views regarding the use of AR technology in CPD?	5) Which part of GP in-service training do you want to focus on, and how is that part related to the government requirements?
6) Which parts of the government requirements should physicians learn?	6) Have you used any kind of technology for learning, and, if so, in what ways have you used it?
	7) What are your views regarding the use of AR technology in CPD?

### Data analysis

The qualitative research software NVivo (version 10, QSR International, Australia) was used as a management and support tool for transcribing, coding, and analyzing the qualitative data. The interview transcripts were analyzed using a thematic inductive analysis approach, which was defined as “a method for identifying, analyzing, and reporting patterns (themes) within data without a preexisting coding frame, or the researcher’s analytic preconceptions” [35]. In line with this method, we performed the following steps:

1) After immersing herself in the interview data by transcribing the interviews verbatim, EZ listened to the recordings several times to make sure that the transcripts were right, read the transcripts several times to become familiar with the data, and took note of initial ideas.
2) EZ produced the initial codes by tagging and labeling meaningful content from the transcripts.
3) EZ grouped the codes into potential categories and themes through discussions with co-authors (UF and ÅS). The categories, themes, and relevant extracts from the transcripts were translated into English for further discussion.
4) EZ reviewed the themes in collaboration with UF and ÅS. New themes were created, and some themes were aggregated while others were discarded. Steps 2, 3 and 4 were iterated several times until themes appeared to form a coherent pattern.
5) We compared the themes to the two research questions and assessed the patterns to shed light on those questions.

### Ethical considerations

Ethical approval (file number S545) was obtained from the Ethics Committee of Tongji Medical College at Huazhong University of Science & Technology (China) in November 2012. All participants were informed about the study’s aim via a written form, which also stated that their participation was voluntary and that they had the right to withdraw at any time. The data were collated and analyzed after their verbal agreement. We considered such verbal consent to be appropriate because of the very low risk associated with participating in this educational study.

## Results

The physicians at the CHCSs in our study are required to provide comprehensive medical services to patients with common and chronic diseases, but, according to the managers, most of these physicians are not fully trained as GPs, although they have gained some relevant competencies during internships in various departments at university hospitals. The participating physicians’ skills continued to improve as they practiced and attended CPD activities that focused on various special subjects.

### Physician demographics

The physicians who participated in the study had a variety of specialties: two certified GPs, five internists, two surgeons, one pediatrician, one dentist, and two specialists in chronic disease management (see Table 2). Eight of these physicians graduated in 2004 or later; six had completed their GP in-service training.

**Table 2:** Physician demographics.

Interviewee	GP Training	Graduated	Current Role	Gender	Type of Medicine^*^
**P1**	No	2008	Internist	Female	Internal
**P2**	Yes	2005	Internist	Female	Clinical
**P3**	Yes	2007	Internist	Female	Integrated traditional Chinese and Western
**P4**	No	2012	Surgeon	Male	Clinical
**P5**	No	1995	Dentist	Female	Oral
**P6**	Yes	Unassigned	GP	Female	General
**P7**	No	1993	Internist	Male	Clinical
**P8**	No	2008	Chronic disease management specialist	Female	Clinical
**P9**	No	2004	Surgeon	Female	Clinical
**P10**	No	2007	Chronic disease management specialist	Female	Clinical
**P11**	Yes	1998	GP	Male	Community
**P12**	Yes	1984	Pediatrician	Female	Clinical
**P13**	Yes	2008	Internist	Female	Clinical

^*^ The interviewees reported the type of medicine that they had most recently studied. For example, P1 and P3 reported the types of medicine from their master’s studies.

### Needs in terms of competence and CPD

Based on the interview transcripts, we identified ten categories and classified them within three themes, as shown in Table 3.

**Table 3:** Categories and themes from the interviews.

Categories	Themes
1. Role and competence requirement 2. Professional-development stage and competence 3. Learning expectations for becoming a qualified GP	Theme 1: The requirements for becoming a qualified GP
4. CPD models 5 Current CPD to become a qualified GP 6. The challenges of using in-service training for GPs	Theme 2: The CPD models and the challenges of CHCSs
7. Previous experience with information and communication technology (ICT)-based CPD 8. Positive attitude toward AR 9. Perceived usefulness of AR-based CPD Negative attitude toward AR	Theme 3: Experiences with ICT-based CPD and acceptance of AR

#### Theme 1: The requirements for becoming a qualified GP

In our interviews, we learned that the physicians who wanted to become qualified GPs were expecting to develop holistic and comprehensive competencies that would differ from those of specialist physicians. The managers stated that their CHCSs needed more GPs due to the communities’ growth. However, not all of the physicians at these CHCSs were qualified GPs, and not all of them had been educated as GPs, either in medical school or in service. The physicians believed that they had much more to learn before becoming a fully trained GP.

Role and competence requirement: The physicians in our study reported that they had to assume the role of a GP at times, especially when on integrated duty, such as during the night shift and when coworkers were on leave. Moreover, they all expected to become qualified GPs. Most of them worked as specialists in the surgery, internal medicine, or dental departments. Although dentists cannot be GPs in most countries, the dentist in this study was working as a GP when we met her in the GP department, and she treated all the patients who came to her CHCS.

The physicians, especially when on duty and when others are not at work, should treat patients, no matter what disease they have. (M1)

However, the physicians also expressed similar ideas even though they were not professional, fully trained GPs. The managers and physicians agreed that GPs should have broad competence:

With the development of the community, more GPs were required. … You know, we do not have certified GPs now. … A GP has to understand everything in medicine but does not have to be proficient in it. (M2)

Physicians in primary care can see any diseases during the first consultation, and our competencies are certainly more comprehensive than those of the specialists. (P8)

Professional development stage and competence: It was also explained that the CHCS physicians had learned about internal medicine, surgery, gynecology, and pediatrics when they studied clinical medicine. However, they had forgotten some aspects of their knowledge and had not continued to improve that knowledge after they graduated because they had mostly worked as specialists; they also expressed that the CHCSs had no senior GPs to help them:

They had to learn all the basics of internal medicine, surgery, gynecology, and pediatrics when they studied clinical medicine. (M2)

In practice, you forget a lot of knowledge if you work as a specialist, as you rarely make use of the other parts of your education over a long time period. (P12)

Learning expectations for becoming a qualified GP: Both groups of interviewees disclosed that the physicians indeed needed CPD to become qualified GPs. The physicians looked forward to continually developing their clinical competence, which includes learning about new guidelines and treatments, as well as (especially) the rational use of medications.

There are many things you need to learn if you want to become a GP because you need to know enough in all areas. It is very broad; we need to learn a lot. … We certainly need to improve our clinical competence and become proficient at diagnosing and treating diseases in the clinic. (P1)

We want to know if there any new treatments, new drugs, or new research advances for use in hospitals, but we do not have access to these advances, and there are very few ways to get access. (P2)

The managers expected the physicians to become fully trained GPs and to learn about both theory and clinical practice. In particular, managers expected the physicians to focus their training on areas that they had not practiced during their time as specialists. They also expected the physicians to develop competence in preventive care, patient education, and chronic disease management.

A physician who is relatively strong in internal medicine needs to improve competence in other subjects. A physician in obstetrics and gynecology should learn internal medicine, surgery, and ear, nose, and throat medicine; I hope to be able to improve my weaknesses. (M1)

[Physicians need to learn] how to deal with clinical problems and then update their knowledge of theory. … Our other major functions are preventive care and health education. We manage high-risk groups … to prevent them from developing chronic diseases. We teach them how to change their lifestyles, to be conscious of and control dangerous conditions so as to prevent, for example, high blood pressure. (M2)

Both managers and physicians reported that the learning should focus on common diseases, prehospital first aid emergencies, and chronic disease management.

At the CHCSs, we need to focus on prehospital and posthospital treatments; we cannot reach the level of specialists in tertiary hospitals through such short-term training. … More often, we learn how to deal with common diseases and health concerns, such as headache, stomachache, high blood pressure, and heart disease. (P2)

At the clinic, we mainly provide prehospital first aid and treat chronic and common diseases. (M2)

#### Theme 2: The CPD models and the challenges of CHCSs

Both the physicians and the managers in our study claimed that physicians have different opportunities to take part in CPD. Whether through their CHCSs or through outside sources, the physicians can learn from lectures in which invited specialists speak about their clinical experiences. Physicians can also join subject-based learning or engage in several-months-long in-service training at a tertiary hospital. Some of the physicians had taken part in other kinds of GP training. In fact, only one of the interviewed physicians reported a GP training that nearly corresponded to the national outline. Most of the GP trainings were shorter and more theoretical than the national requirements. Another challenge that the physicians faced was a lack of opportunities to take part in in-service GP training.

CPD models: The most common type of CPD for the interviewees was lecture-based trainings hosted by the Health Bureau of Wuhan, their own CHCSs, or academic conferences. Moreover, one manager mentioned a presentation from pharmaceutical agents as a potential CPD activity. The CPD lectures’ topics were mostly broad, so they could enhance the physicians’ knowledge. However, some lectures went deeper to explain special diseases; the physicians regarded these lectures as being of no use.

I learned through outside training, which consisted of two- to three-hour lectures and by participating in some conferences. (P9)

A number of pharmaceutical companies and pharmaceutical agents provided lectures on the respiratory, digestive, and endocrine systems. … The topics of the lectures are very much connected to real life–for example, new developments in clinical applications for diabetes and hypertension. (M1)

Some of the physicians had joined subject-based learning activities on various topics according to their current roles at the CHCSs. These learning activities were designed to focus on the main health care tasks that the physicians were expected to handle. This kind of CPD was in-practice training and was best suited to specialists.

I participated in some training on first aid operations in emergencies. … The main clinical work I faced was providing first aid in emergencies: emergency operations, gastric lavage, inspiratory suction, etc. (P4)

In this period–for example, during the simulation of military training for new students at the university–we arranged first aid education … for diseases such as heat stroke and acute gastroenteritis. (M2)

In our study, some of the physicians reported that they engaged in a several-months-long in-service training at a tertiary hospital to improve their competence as specialists, but this training was mainly for young physicians.

I was a pediatrician because I was in the department of pediatrics. Mostly, I treated pediatric diseases. Then, we took part in a short-term in-service training. This training was still focused on pediatrics. (P12)

This in-service training is hard for the senior physicians because it is for first-line clinical physicians at a tertiary hospital. They cannot take be first-line physicians because of their age. (M1)

The CPD models are summarized in Table 4.

**Table 4:** Summary of the CPD models.

CPD Method	CPD Topic Examples	CPD Host	Teacher	Benefits for a GP	Limitation
Lecture	Cardiovascular diseases Common childhood illnesses Anesthesia Age-related illnesses Prescription comments Diabetes Hypertension Respiratory diseases Digestive diseases Endocrine diseases	The Wuhan Health Bureau Tertiary hospitals	Specialists	These are common patient problem; this CPD is useful for decisions related to referrals or management.	Some lectures, such as those on serious cardiac disease, are not useful for GPs. The lectures are not part of the systematic GP training.
		Pharmaceutical companies	Pharmaceutical agents	Physicians can update their knowledge of new drugs and treatments, side effects, etc. This is mutually beneficial for the physician and the company.	The pharmaceutical company may expect the physicians to prescribe expensive drugs.
Subject-based learning	First aid operations Acute gastroenteritis	CHCS	Senior physicians	Physicians can learn practical skills.	This training is only for physicians who work in emergency care.
In-service training for specialists	Cardiovascular diseases Pediatric diseases	Tertiary hospitals	Specialists	The physicians can gain familiarity with how to manage diseases.	This is part of training for a specialist; it is not suitable for senior physicians.

Current CPD to become a qualified GP: Some of the physicians in the study had taken part in several kinds of GP training activities, and they explained that the trainings varied in terms of time, learning method, and content. They reported that their GP CPD was based only on lectures and lacked practice, or that it was focused on theoretical learning with only limited clinical practice. One physician said that he had joined an eleven-month in-service training that included the stipulated ten months of clinical practice in a tertiary hospital and one month of theoretical learning. During the ten months of clinical practice, he moved between departments (emergency, neurology, cardiology, gastroenterology, etc.), studying for one month in each. Other physicians reported that they had participated in community medicine lectures that could be part of the GP training.

I participated in GP training. … It was five months of training, including face-to-face teaching for more than three months and practice training for more than one month. (P3)

The GP training is one to two months of intensive courses or two to three months of weekend training in a high-level hospital. When this kind of training is completed, the physicians complete a simple internship practice in a good CHCS. (M1)

The challenges of using in-service training for GPs: The first challenge that the interviewees reported was that physicians did not always have a chance to accept government-offered GP training because their managers’ plans did not consider the number of physicians on duty at the CHCS. In addition, physicians who had been more recently hired at their CHCSs had fewer opportunities. These opportunities could also be affected by their managers’ attitudes toward the GP training.

The GP training needs to be outside of working hours. We cannot work in the hospital [during the training, so] we have to take turns. (P4)

I have not been able to do the GP training because I have worked in this CHCS for only a short period of time. (P7)

We did not make plans. The health bureau will give us the index of health staff for GP training every year. We can follow this index to arrange for the physicians to take part in the training. (M1)

The second challenge was the poor training efficacy, as physicians who had the chance to take part in the GP training complained about its limited effects. They made specific complaints that only large hospitals provided the training and that it did not address the needs of the CHCSs. Although the standards of the national training were applied, the training did not consider the differences between hospitals. The managers reported that the current GP training was not sufficient for their CHCSs.

I would rather read by myself than listen to the teacher talking. What the teacher told us is exactly the same as what you can learn from the book. (P2)

It (the GP training) included theoretical as well as practical training, and it was quite serious. Anyway, it had an effect, but we could not entirely depend on it. (M 2)

#### Theme 3: Experiences with ICT-based CPD and acceptance of AR

In the interviews, both of the managers and most of the physicians expressed positive attitudes toward the use of AR for CPD. Those who had a positive attitude thought that AR could be especially useful for learning and training related to the nervous system, certain types of surgery, emergency care, antibiotic treatment, and patient safety. Moreover, both groups also suggested that smartphones could be a useful tool for AR applications.

Previous experience with ICT-based CPD: The physicians reported that they used ICT to update their knowledge, mainly through online searches, social media, and virtual communities for clinicians. They searched for online guidelines or electronically published papers from medical journals or newsletters to update their knowledge. The physicians perceived that this type of search was convenient and that it reduced memory load. They also reported that they received (and shared) information with their friends, classmates, colleagues, and supervisors through social media (e.g., WeChat and QQ). In addition, the physicians reported that they read articles or asked questions in virtual communities for physicians (e.g., DingXiang Yuan, which was developed for all levels of physicians).

I often search online when I face a confusing situation; we cannot remember all the information that we have learned. (P12)

Most of our friends and colleagues are physicians. We share knowledge on WeChat. (P9)

We physicians often use a website called DingXiang Yuan. (P13)

Although the physicians said that ICT provided flexibility and convenience for CPD, they also mentioned some challenges of ICT use, such as the fact that learning with ICT was dependent on the individual physician’s initiative. Moreover, they reported that determining the accuracy and relevance of information from various sources was challenging.

The authority (of information online) cannot be guaranteed. This is only to say that we get general information. For work in the hospital, we need specific information, and everything should be clear. (P12)

Positive attitude toward AR: Both of the managers and most of the physicians expressed a positive attitude toward AR. The managers reported that AR-based CPD could be convenient for the physicians, who preferred learning in the workplace. The main positive comments regarding AR were that it seemed convenient, visual, and impressive. Based on their positive attitudes, the managers and the physicians suggested that AR could be developed for smartphones. Moreover, the physicians suggested that learning activities be systematically organized and that AR courseware be developed specifically for them.

It would be great if you could systematically organize the learning activities. … It would also be great if you could develop such good courseware. … It looks like a simulation of human body, and it is very realistic; it could be very useful but very expensive. (P1)

Could it be applied to different computers? Everyone has a computer, but some people may not be able to access the internet because that would affect the operations of the hospital system. Every physician has a smartphone with which they could access the internet, however. (M2)

Perceived usefulness of AR-based CPD: The physicians regarded the 3D visualization that they saw as a possibility for improving CPD for GPs, as it gave them a better understanding of human physiology; they also thought it would be good at helping them to remember details. They said that AR would be good for motivating GPs and making them more interested in learning. One physician even reported that learning with AR could be effective at improving patient safety and that it could be beneficial for future medical students. Furthermore, it was suggested that AR could be more useful if was developed for certain topics. They mentioned that it could be used to learn about various internal body systems and processes that cannot be seen clearly (e.g., the nervous system and antibiotic treatment). The physicians also thought that AR would be meaningful for surgery and emergency care, which need hands-on operational skills.

It would be very good if you could develop this really well. There are many medical operations, yeah, such as cardiopulmonary resuscitation and tracheal intubation; there are a lot of things we need to learn. (P1)

This is certainly better; could you develop other systems like this? I am sure that I would like to use this, especially when to learn about the nervous system. (P7)

This is better, for example, when the organs are infected and need to be treated with antibiotics. I think that it could be used for pulmonary, intestinal, and other infections. We can see it clearly. (P10)

Negative attitude to AR: Only one physician expressed doubt regarding the effectiveness of AR; this doubt was based on her learning experiences with mannequins in medical school.

There is a difference between how we do it and how we see it. It is much different. We practiced on mannequins instead of real people when we studied at university, and it was a big difference when we later did it on real patients. It would also be different if the training is supported by computers because you do not have the real stuff. (P5)

## Discussion

In this study, we investigated physicians’ need for CPD in light of the requirements for becoming qualified GPs, as well as the possibility of using AR for this purpose as part of a Chinese primary health care setting. The physicians and managers agreed on the necessity of supplying CPD activities that are aimed at helping physicians to become fully trained GPs and at ensuring that their competencies are different from those of specialists. Most of the CHCS physicians in the study did not take part in GP training during medical school, but we found that they did not regard the current forms of CPD training as adequate for physicians who want to become qualified GPs. The managers and physicians reported that AR could support a good learning method because they perceived it as convenient, visual, and impressive. They provided more suggestions for AR development that could make the design more flexible to ensure that AR meets their needs.

### The competence gap among physicians who are becoming qualified GPs at CHCSs

The managers in our study mentioned that they needed more qualified GPs and that the vast majority of the current physicians at their CHCSs were not trained as GPs. This finding was confirmed by a systematic literature review of the CHCS development [7]. The physicians in this study mainly worked as specialists after graduation, and they needed to improve their clinical competence as GPs; the national outline for GP in-service training also emphasized this need [8]. Moreover, the physicians reported that CHCSs should mainly provide comprehensive services, with a special focus on integrating diagnosis and treatment. Such integrated service could be related to the GPs’ core competencies and should have a comprehensive approach, a community orientation, and use holistic modeling, as in the European Union [36]. However, the Chinese physicians in this study did not mention the other core competencies of European GPs, such as primary care management, person-centered care, and specific problem-solving skills. A Chinese GP’s integrated-services competence is expected to include “prevention and health care; diagnosis, treatment, and referral of common diseases; rehabilitation and management of chronic diseases; and health management”[4]–some of which the physicians in our study did not cite. The physicians did acknowledge that competence in the diagnosis and treatment of common or chronic diseases was important, but they overlooked prevention and management. They may have done so because they do not yet truly work as GPs or because traditional health care education focuses more on basic biomedicine, medical technology, and clinical medicine than on prevention and management [37].

### The CPD learning methods

The interviewees reported challenges related to GP training, including the lack of opportunities to take GP in-service training courses and the inadequate effects of the current GP training. The poor efficacy of the GP training has been previously reported [3,38,39].

Technologies were suggested to be used for improving the accessibility of CPD [15]. Our study indicates, first, that physicians use the internet to search for and share information and, second, that they question the accuracy and relevance of some online resources. The physicians in our study also revealed that they use virtual communities to get in touch with other physicians. Other scholars have explored this kind of ICT usage and shown that it enhances health care professionals’ development through “communities of practice,” in which health care professionals can come together and share experiences and ideas so that they can be innovative together [40]. Moreover, previous researchers have shown that, even though ICT provides learning resources for GPs’ CPD, barriers remain in ensuring that it is effectively used [41] and in designing it to meet the required educational needs [42]. CPD learning modules that combine online and face-to-face activities need to be of a blended nature [43].

In this study, we did not attempt to compare the kinds of technology or methods that can be used in CPD. Instead, we tried to understand the needs of CHCSs and the possibilities available technology. It was shown that the physicians in our study already use different kinds of ICT for CPD. When introduced to AR, the managers and most of the physicians were positive about its use in future CPD. This positive attitude was also found in other studies [25,44]. Moreover, the participants believe that AR would be useful for future CPD, especially for topics concerning internal body systems (which cannot be easily seen), as well as for hands-on skills training. In contrast to other researchers, who have evaluated the acceptance of existing AR systems, we explored the possibility of using AR as an initial step in a future design of CPD for GPs.

### Comparison with other studies

In this study, we analyzed physicians’ CPD needs; this constitutes the initial step toward providing an effective AR-based CPD for Chinese primary care physicians. Grant (2012) also considered this initial analysis of CPD. Evaluating physicians’ personal views and their previous CPD experiences is one way of conducting a CPD needs analysis [13]. Scholars have also used qualitative studies to understand both CPD needs and the challenges of offering CPD in a primary care setting [45,46]. Unlike in the prior studies, which focused on other health care professionals (such as physiotherapists) and on nonmedical prescribing, we explored physicians’ needs regarding general competence in the primary care setting. Moreover, we provided views from managers, whose overall support is an important factor in effective CPD for GPs [14]. A Malaysian study revealed that the same common CPD methods as in this study, including conferences, lectures, and online recourses; the GPs in that study also desired a more effective method [47]. A Chinese study in a rural primary health care setting also indicated that face-to-face CPD was ineffective, and the physicians in that study expressed demand for novel methods of increasing quality and accessibility [48]. Their results showed that health care workers had positive attitudes toward web-based learning, even though there were obstacles such as low computer literacy and a lack of time and motivation. In addition, our study showed that physicians and managers had positive attitudes toward AR-based CPD and believed that it would contribute to useful learning methods.

### Implications for design

To design effective CPD applications, it is necessary to help GPs improve their competencies, to understand their learning needs, and to stimulate positive attitudes toward technology. The designers of such applications should consider the context of professionals, patients, and the national health care system [49]. Our results revealed that both managers and the physicians themselves considered physicians’ CPD to be necessary.

Inspired by the interviewees’ positive attitudes toward AR, we believe that applying learner-centered design to AR applications could lead to better opportunities for learning in the workplace. An AR-based CPD design should improve ease of use and usefulness in accordance with Davis’s technology acceptance model, which demonstrates that the actual use of a technology relies on the user’s attitude toward that technology, its perceived usefulness, and its perceived ease of use [26]. Moreover, our interviewees considered AR to be visual and impressive, so that technology might motivate them to learn to become a qualified GP. Moreover, as AR could also provide physicians with authentic and situated experiences within their clinical environments, it could facilitate their expected practice-oriented and case-based learning. Smartphones could be a good platform (according to their suggestions) because most physicians in China have access to such devices [50].

Although many aspects are required when learning to become a qualified GP, different physicians and CHCSs have distinct needs. Flexibility is one design concern–whether it is the design of physical products, organizations, or learning- and knowledge-management systems [51]. Regarding CPD for GPs in CHCSs, the design of learning tools should be flexible and should find a balance between the physicians’ learning needs (based on their competence gaps) and the tools that they suggest should be developed. In addition, the design should also consider the balance between individual needs and common needs (e.g., tools that other GP groups can use).

### Strengths and limitations

In this study, we have explored the current status of CPD and the potential value of AR in providing CPD for GPs as part of China’s complex health care system. Interviews with physicians and managers at four CHCSs in Wuhan, China, (during a period of months across different years) provide data, space, and time triangulation [52]. Triangulation can improve the credibility of this study [34]. This study focused on the Chinese primary health care situation, and the results might not be applicable in other contexts; however, it could be useful in similar situations where the specialists will be trained to become qualified GPs.

There are several limitations that need to be pointed out. First, there are many ways of discovering CPD learning needs. We focused on interviews with physicians and managers to provide a comprehensive delineation that could be interesting for other researchers and for policy makers. The study focused on general concerns of the physicians and did not cover details about their specific learning needs. Second, we tried to involve all the stakeholders relative to CHCSs but did not involve medical teachers, as there are only a few GP teachers in Wuhan. We also need to consider the limitation of the convenience sampling strategy to find respondents, which might limit the generalizability of our results. Furthermore, only the first author is native Chinese, which might limit at the reliability of the transcriptions and translations. However, we see this minor issue because the whole research team has substantially discussed all of the translated material.

## Conclusions and suggestions for further work

In our empirical study in Wuhan, China, both physicians and managers in primary care expressed that they wanted practicing physicians to improve their clinical competence regarding common and chronic diseases. This is also required by the Chinese government. However, the CPD training activities that our study participants had experience of did not meet these needs. Furthermore, not all physicians got the chance to join the GP in-service training that is required by the government. There were also physicians who complained about the effect of the current GP training in which they had participated. Physicians in the study exemplified how AR could be useful for learning, especially for some topics such as the nervous system, surgery, emergency care, and antibiotics treatment. Integrated clinical competence regarding common and chronic diseases could be a priority when designing new CPD training using AR.

As a first step in designing effective CPD activities, this study focused on necessity and possibility. The next step should focus on learning methods and the design of a working AR-based CPD training program that can be put into practice. We need to explore models that can guide us to the development of AR support for CPD that could meet our physicians’ learning needs. In connection with this, we also need to further explore the common problems of GPs’ clinical competence.
